# Real-time Imaging Orientation Determination System to Verify Imaging Polarization Navigation Algorithm

**DOI:** 10.3390/s16020144

**Published:** 2016-01-23

**Authors:** Hao Lu, Kaichun Zhao, Xiaochu Wang, Zheng You, Kaoli Huang

**Affiliations:** 1 State Key Laboratory of Precision Measurement Technology and Instruments, Tsinghua University, Beijing 100084, China; luh14@mails.tsinghua.edu.cn; 2 Ordnance Engineering College, Shijiazhuang 050003, China; ddgcx@163.com; 3 Qian Xuesen Laboratory of Space Technology, China Academy of Space Technology, Beijing 100094, China; wangxiaochu1985@gmail.com

**Keywords:** orientation determination, Zhang’s calibration, multi-camera imaging polarimeter, bio-inspired polarization navigation

## Abstract

Bio-inspired imaging polarization navigation which can provide navigation information and is capable of sensing polarization information has advantages of high-precision and anti-interference over polarization navigation sensors that use photodiodes. Although all types of imaging polarimeters exist, they may not qualify for the research on the imaging polarization navigation algorithm. To verify the algorithm, a real-time imaging orientation determination system was designed and implemented. Essential calibration procedures for the type of system that contained camera parameter calibration and the inconsistency of complementary metal oxide semiconductor calibration were discussed, designed, and implemented. Calibration results were used to undistort and rectify the multi-camera system. An orientation determination experiment was conducted. The results indicated that the system could acquire and compute the polarized skylight images throughout the calibrations and resolve orientation by the algorithm to verify in real-time. An orientation determination algorithm based on image processing was tested on the system. The performance and properties of the algorithm were evaluated. The rate of the algorithm was over 1 Hz, the error was over 0.313°, and the population standard deviation was 0.148° without any data filter.

## 1. Introduction

Bio-inspired polarization navigation is a novel, promising navigation method. When studying the foraging behavior of insects, researchers have discovered that insects treat the polarization pattern of scattered skylight as an azimuth reference [1,2]. Several prototypes have been developed based on the polarization-sensitive structure of insects [3,4,5,6,7,8]. These prototypes use photodiodes as photoelectric converters so that a decline occurs when the beams received by the sensors are shrouded by shelters, such as clouds. Imaging polarization navigation sensors can solve these problems because they can obtain abundant polarization information as well as spatial information with high precision and strong anti-interference. Few appropriate imaging polarimeters for navigation have been tested, although recent algorithms have been proposed that extract navigation information from the image of polarized skylight [9,10].

Developing an imaging polarimeter is essential to test and verify the imaging polarization navigation algorithm, although many types of imaging polarimeters are available for measuring the radiance distribution in sky or ocean [11,12,13,14,15,16] or for remote sensing. These instruments or systems are unsuitable to verify the navigation algorithm on account of their high complexity, cost, or insufficiency in real-time applications. An instrument with a rotating element is the imaging polarimeter that is easiest to build, but it does not meet the requirements in real-time because images for calculation are not acquired simultaneously [9,10,12,13,15]. The instruments for measuring radiance distribution in the sky and ocean share the similarity of having three cameras. Large field-of-view fisheye lenses can acquire considerable information, but it increases the probability for sunlight to enter the camera. The fisheye camera system needs high-precision assembly and highly complex calibration procedures [16] than a general wide-angle lens. The prototype consists of a liquid crystal variable retarder that is costly and runs under strict thermal conditions [14]; by contrast, the imaging polarimeter often runs under uncertain thermal conditions.

In this study, an easy-to-build real-time imaging orientation determination system was designed and implemented to verify the imaging polarization navigation algorithm. The optical system contained three 8 mm lenses to avoid received sunlight and for ease in calibration and rectification. The essential calibration procedures for this type of system, such as camera parameter calibration, and the inconsistency of complementary metal oxide semiconductor (CMOS) calibration were analyzed, designed, and implemented. The results were used to undistort and rectify the multi-camera system. The results of the orientation determination experiment indicated that the system could acquire and compute the polarized skylight images, while the imaging polarization navigation algorithm described in [9] to be tested was loaded in the system, which can resolve orientation in real-time. The update rate of the system was over 1 Hz, the error was over 0.313°, and the population standard deviation was 0.148° without any data filter.

## 2. Fundamentals of Instrument Description

### 2.1. Principle of the System

The fundamentals of the polarization imaging algorithm of the system are based on the Stokes parameter method, which can describe the polarization state of electromagnetic radiation through light intensity. The Stokes parameter contains four parameters, which are *I*, *Q*, *U*, and *V*. *V* is deduced because circular light hardly exists in skylight [17,18,19]. The Stokes vector is defined as $S={\left[\begin{array}{ccc} I & Q & U \end{array}\right]}^{T}$ in this study. Mueller calculus is a mathematical operation that describes the effect of lenses or polarizers on a beam, which can be expressed by manipulating the Stokes vectors as:

(1) $$S_{out}=MS_{in}$$

The first row of the Mueller matrix is extracted because the only variable that can be measured directly is $I_{out}$ in $S_{out}$, *i.e.*,:

(2) $$I_{out}=m_{11}I_{in}+m_{12}Q_{in}+m_{13}U_{in}\to I_{out}=\frac{1}{2}\left(I_{in}+Q_{in}\cos2\alpha +U_{in}\sin2\alpha \right)$$

where *α* is the angle of the axis of the polarizer from x–axis of the coordinates we define. The x–axis of the coordinates points to the right of the camera and the y–axis points to the bottom as viewed from the lens. The system comprises three cameras, namely, A, B, and C. Three polarizers with axes of −90, −45, and 0 exist in front of the cameras.

(3) $$\left[\begin{array}{c} I_{-90}\left(i,j\right)\\ I_{-45}\left(i,j\right)\\ I_{0}\left(i,j\right) \end{array}\right]=\frac{1}{2}\left[\begin{array}{ccc} 1 & -1 & 0\\ 1 & 0 & -1\\ 1 & 1 & 0 \end{array}\right]\left[\begin{array}{c} I\left(i,j\right)\\ Q\left(i,j\right)\\ U\left(i,j\right) \end{array}\right]$$

According to Equation (3), ***I***, ***Q***, and ***U*** images are linearly transformed from intensity images ***I***_−90_, ***I***_−45_, and ***I***_0_ gathered by cameras A, B, and C, respectively, as:

(4) $$\left[\begin{array}{c} I_{-90}\left(i,j\right)\\ I_{-45}\left(i,j\right)\\ I_{0}\left(i,j\right) \end{array}\right]=\frac{1}{2}\left[\begin{array}{ccc} 1 & -1 & 0\\ 1 & 0 & -1\\ 1 & 1 & 0 \end{array}\right]\left[\begin{array}{c} I\left(i,j\right)\\ Q\left(i,j\right)\\ U\left(i,j\right) \end{array}\right]\underset{inv}{\to }\left[\begin{array}{c} I\left(i,j\right)\\ Q\left(i,j\right)\\ U\left(i,j\right) \end{array}\right]=\left[\begin{array}{ccc} 1 & 0 & 1\\ -1 & 0 & 1\\ 1 & -2 & 1 \end{array}\right]\left[\begin{array}{c} I_{-90}\left(i,j\right)\\ I_{-45}\left(i,j\right)\\ I_{0}\left(i,j\right) \end{array}\right]$$

The angle of polarization (***AoP***) and the degree of linear polarization (***DoLP***) images are calculated from ***I***, ***Q***, and ***U*** images as follows [20]:

(5) $$AoP^{\prime }\left(i,j\right)=\{\begin{array}{cc} \frac{1}{2}\arctan\left(\frac{U\left(i,j\right)}{Q\left(i,j\right)}\right) & Q\left(i,j\right)<0\\ \frac{1}{2}\arctan\left(\frac{U\left(i,j\right)}{Q\left(i,j\right)}\right)+90^{\circ } & \left(Q\left(i,j\right)>0\right)\wedge \left(U\left(i,j\right)<0\right)\\ \frac{1}{2}\arctan\left(\frac{U\left(i,j\right)}{Q\left(i,j\right)}\right)-90^{\circ } & \left(Q\left(i,j\right)>0\right)\wedge \left(U\left(i,j\right)>0\right) \end{array}$$

(6) $$DoLP\left(i,j\right)=\frac{\sqrt{U^{2}\left(i,j\right)+Q^{2}\left(i,j\right)}}{I\left(i,j\right)}$$

We define the image of the angle of the orientation of the E-vector as the ***AoE*** image, and the angle is from the local meridian:

(7) $$AoE\left(i,j\right)=AoP^{\prime }\left(i,j\right)-\varphi$$

where φ is the angle of local meridian clockwise from y–axis of the coordinates we define as viewed from the lens.

### 2.2. Structure of the System

**Figure 1:**
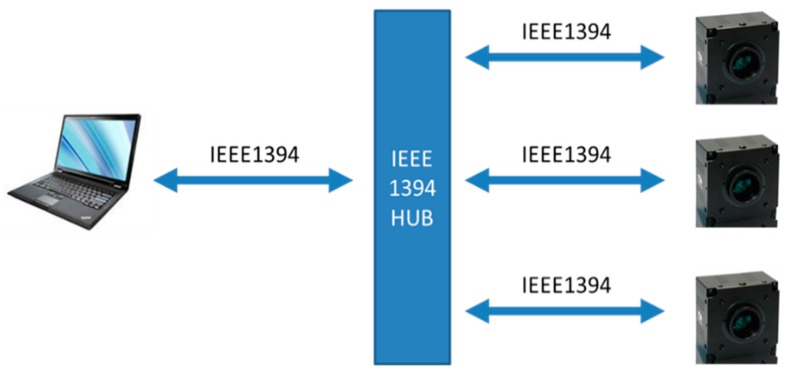
Hardware structure of the system.

The multi-camera system has the advantage of simultaneous imaging. In this study, a fisheye lens is an unnecessary option because the system is not designed to acquire as much data as possible. The optical system contains three Computar m 0814-mp 8 mm lenses (Computar, Tokyo, Japan), and they are mounted on three Daheng HV1310-FM monochrome cameras (Daheng, Beijing, China). The three cameras are bolted on an aluminum structure. The data acquired by the cameras are transferred through the IEEE-1394 bus and processed and calculated on a laptop. The three IEEE-1394 buses from the three cameras are concentrated by a hub to one bus for a single port on the computer, as shown in Figure 1.

**Figure 2:**
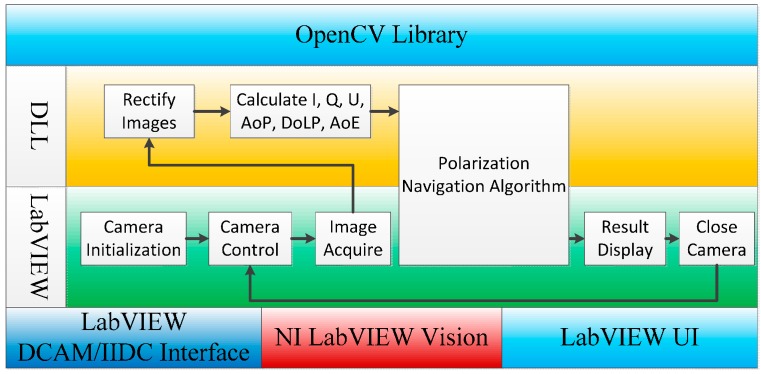
Flowchart of the primary process program.

Several subprograms exist in the main program, which are the image acquisition program, the camera control program, the user interface program, and the process program which contains the polarization navigation algorithm to be tested. The main program, and most subprograms, are programmed in LabVIEW. Off-the-shelf interfaces exist for instrumentation and industrial digital camera specification (IIDC) IEEE-1394 camera to control and acquire data. The process program in which the ***AoP*** image and the ***DoLP*** are calculated from intensity images, the corrections of cameras, and the pattern recognition code are developed in the C programming language and packaged in a dynamic link library (DLL) that can be called by LabVIEW, as presented in Figure 2.

## 3. Calibration

### 3.1. Calibration of Camera Parameters

**Figure 3:**
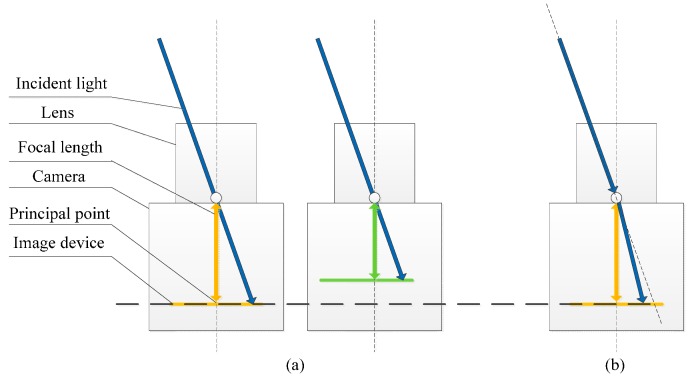
(**a**) Inconsistency caused by intrinsic parameters; and (**b**) inconsistency caused by the distortion of lens.

**Figure 4:**
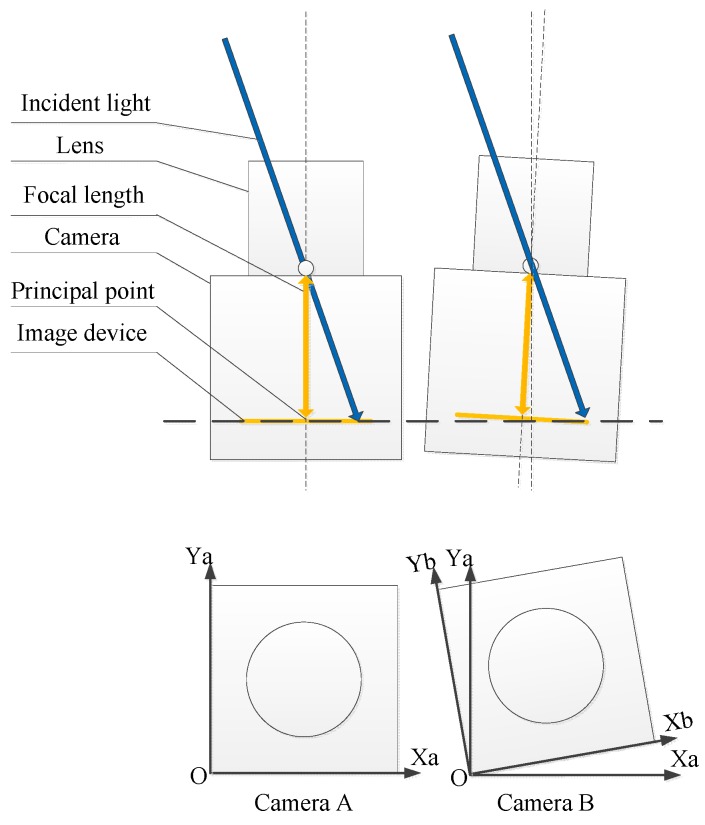
Inconsistency caused by extrinsic parameters.

The general calibration procedures, which are roll off, linearity, dark offset, and immersion calibrations for the camera system, were discussed in [21,22,23]. In this study, we concentrate on the critical methods in calibration for the multi-camera system because inevitable inconsistencies occur, namely, the inconsistency in the intrinsic and extrinsic parameters and the response inconsistency of CMOS. A hypothesis is established for the system to correctly run such that the cameras acquire the same images if no analyzer exists in front of the cameras. In fact, the inconsistency of intrinsic parameters is caused by the manufacturing error of lens and the assembly of CMOS and lens, as shown in Figure 3. The inconsistency of extrinsic parameters is caused by the assembly of cameras, as shown in Figure 4. The inconsistency of cameras cannot be diminished, although we can achieve an accurate assembly, which is difficult and expensive to create. Nonetheless, the inconsistency of cameras can be calibrated and compensated easily.

The pin-hole camera model can be expressed as:

(8) sm′=Α[R|t]M′

or:

(9) $$s\left[\begin{array}{c} u\\ v\\ 1 \end{array}\right]=\left[\begin{array}{ccc} f_{x} & 0 & c_{x}\\ 0 & f_{y} & c_{y}\\ 0 & 0 & 1 \end{array}\right]\left[\begin{array}{cccc} r_{11} & r_{12} & r_{13} & t_{1}\\ r_{21} & r_{22} & r_{23} & t_{2}\\ r_{31} & r_{32} & r_{33} & t_{3} \end{array}\right]\left[\begin{array}{c} X\\ Y\\ Z\\ 1 \end{array}\right]$$

where (X,Y,Z) are the coordinates of a 3D point in the world coordinate space. (u,v) are the coordinates of the projection point in pixels. ***A*** is a camera matrix or an intrinsic parameter matrix. $\left(c_{x},c_{y}\right)$ are the principal point. $\left(f_{x},f_{y}\right)$ are the focal lengths. *s* is a factor scale that is often equal to *Z*.

(10) $$\left[\begin{array}{c} X\\ Y\\ Z \end{array}\right]=\left[\begin{array}{c} r\sin\theta \cos\varphi \\ r\sin\theta \sin\varphi \\ r\cos\theta \end{array}\right]$$

where (*r*, *θ*, *φ*) are the spherical coordinates of points in the world coordinate space. The spherical coordinates are appropriate to describe the skylight beams received by the system. Substituting Equation (10) into Equation (8) or (9), we obtain the following:

(11) $$s\left[\begin{array}{c} u\\ v\\ 1 \end{array}\right]=\left[\begin{array}{ccc} f_{x} & 0 & c_{x}\\ 0 & f_{y} & c_{y}\\ 0 & 0 & 1 \end{array}\right]\left[\begin{array}{cccc} r_{11} & r_{12} & r_{13} & t_{1}\\ r_{21} & r_{22} & r_{23} & t_{2}\\ r_{31} & r_{32} & r_{33} & t_{3} \end{array}\right]\left[\begin{array}{c} r\sin\theta \cos\varphi \\ r\sin\theta \sin\varphi \\ r\cos\theta \\ 1 \end{array}\right]$$

Many methods can be used to calibrate cameras, such as Zhang’s method [24]. This calibration method only requires cameras to observe a painted planar pattern shown at a few different orientations. Either cameras or the planar pattern can be freely moved. In the present study, the intrinsic parameter matrix ***A*** and the extrinsic parameter matrix ***R*** are essential, whereas the translation vector can be neglected because the offset of the same beam caused by the translation among the three cameras (approximately 50 mm) is less than 1 pixel (8 mm lens is selected in the system). The calibration precision is approximately several pixels.

In the past, Zhang’s calibration was always used for single-camera calibration or for stereo calibration. To calibrate a triple-camera system, we divide the calibration into three single-camera calibrations and two stereo calibrations. First, several groups (approximately 20 groups) of images are acquired for calibration. Photos of the same planar pattern for each group should be taken simultaneously, and all grids on the plane should be in the field of view of all cameras. Second, the intrinsic matrices of cameras A, B, and C should be calculated separately, and the calibration results should be saved. Finally, the extrinsic matrices should be calibrated. In this step, two stereo calibrations between cameras A and B and between cameras A and C are conducted because the coordinate space of camera A is selected as the world coordinate space. The result of this step is expressed as two Rodrigues vectors that indicate the relativity of the coordinates of cameras B and C with respect to the coordinates of camera A.

### 3.2. Calibration of Inconsistency of Cameras

We can consider that respective pixels (*i, j*) of cameras measure identical beams geometrically after the calibration in Section 3.1. The responses of the respective pixels (*i, j*) of cameras should be identical according to Equation (4). The model of the response of cameras can be expressed as [23]:

(12) $$\begin{array}{c} I_{-90}\left(i,j\right)=Ka\left(i,j\right)I_{A}\left(i,j\right)\\ I_{-45}\left(i,j\right)=Kb\left(i,j\right)I_{B}\left(i,j\right)\\ I_{0}\left(i,j\right)=Kc\left(i,j\right)I_{C}\left(i,j\right) \end{array}$$

***I***_A_, ***I***_B_, and ***I***_C_ denote geometric calibrated images without the dark offset acquired by cameras A, B, and C. ***I***_−90_, ***I***_−45_, and ***I***_0_ are the ideal images for polarization calculation. The necessary coefficient images ***Ka***, ***Kb***, and ***K*c** should be involved to compensate for the inconsistency of cameras. A large stable dome source is an ideal data source for the system, although it is infeasible. Nonetheless, we can regard skylight as the “dome” under good weather conditions. When calibrating, filters should be removed and cameras should shoot the same scene, such as the “dome”. Images sequences $I_{A}\left(k\right)$, $I_{B}\left(k\right)$, and $I_{C}\left(k\right)$ are collected, rectified. Averaged images $I_{A}^{mean}$, $I_{B}^{mean}$, and $I_{C}^{mean}$ are calculated as Equation (13) to decrease the effect of the instability of skylight and the random noise of cameras:

(13) $$I_{A}^{mean}=\frac{\sum_{k}I_{A}\left(k\right)}{N},I_{B}^{mean}=\frac{\sum_{k}I_{B}\left(k\right)}{N},\mathrm{and}I_{C}^{mean}=\frac{\sum_{k}I_{C}\left(k\right)}{N}$$

The coefficient images ***Ka***, ***Kb***, and ***Kc*** are calculated as Equation (14):

(14) $$\begin{array}{l} I^{mean}=\frac{I_{A}^{mean}+I_{B}^{mean}+I_{C}^{mean}}{3}\\ Ka\left(i,j\right)=\frac{I^{mean}\left(i,j\right)}{I_{A}^{mean}\left(i,j\right)}\\ Kb\left(i,j\right)=\frac{I^{mean}\left(i,j\right)}{I_{B}^{mean}\left(i,j\right)}\\ Kc\left(i,j\right)=\frac{I^{mean}\left(i,j\right)}{I_{C}^{mean}\left(i,j\right)} \end{array}$$

### 3.3. Correction

When the system runs, the original images acquired by cameras A, B, and C are remapped using their intrinsic matrices to rectify the images. The rectified images of cameras B and C are then remapped to the coordinate of camera A, and the three images should be in the same coordinates. Finally, the geometrically-transformed images are multiplied by coefficient images ***Ka***, ***Kb***, and ***Kc*** as Equation (12), and qualified to calculate Stokes images.

## 4. Polarization Navigation Algorithm

The system is designed to verify the algorithm of the imaging polarization navigation algorithm. In this study, the orientation algorithm based on the image processing is an angle algorithm of which the azimuth refers to the solar meridian. The solar meridian has three features, namely, an E-vector of 90°, a straight line, and through the principal point. These three features are sufficient conditions to define a line as the solar meridian [9]. Thus, the algorithm consists of threshold extraction and a special simplified Hough transform (HT).

The threshold extraction is expressed as follows:

(15) $$\left\{ \begin{array}{l} BSM\left(i,j\right)=1,\left(\left|AoE\left(i,j\right)-\frac{\pi }{2}\right|<R\right)\\ BSM\left(i,j\right)=0,\left(\mathrm{the}\;\mathrm{others}\right) \end{array}\right.$$

where ***BSM*** is the binary image of the solar meridian. Owing to the noise, no exact 90° point exists, and tolerance *R* is needed. The principle of the selection of *R* is discussed in [9]. The binary points in *BSM* are transformed to a parameter space by the HT as Equation (16). The peak in the parameter space stands for the solar meridian, and the coordinate of the peak is the parameter of the solar meridian.

(16) $$H\left(\Omega \right)=\sum_{i=1}^{n}k\left(X_{i},\Omega \right)$$

where $X_{i}=\left[\begin{array}{cccc} x_{1i} & x_{2i} & \ldots & x_{Ni} \end{array}\right]$ denotes the feature points defined in an N-dimensional feature space, and *i* is the index of feature points. $\Omega =\left[\begin{array}{cccc} \omega_{1} & \omega_{2} & \ldots & \omega_{M} \end{array}\right]$ is a point in an M-dimensional parameter space. The function *k* is the HT kernel. In the system, the line to measure is in a 2D feature space, and the variable of the line to measure is only the angle *θ* that goes through the principal point. Thus, the HT in the algorithm can be expressed as:

(17) $$H\left(\theta \right)=\sum_{i=1}^{n}k\left({\left(x,y\right)}_{i},\theta \right)$$

The kernel function is as follows:

(18) $$k\left({\left(x,y\right)}_{i},\theta \right)=\{\begin{array}{c} 1\;\tan\theta =\frac{y}{x}\\ 0\;\mathrm{others} \end{array}$$

**Figure 5:**
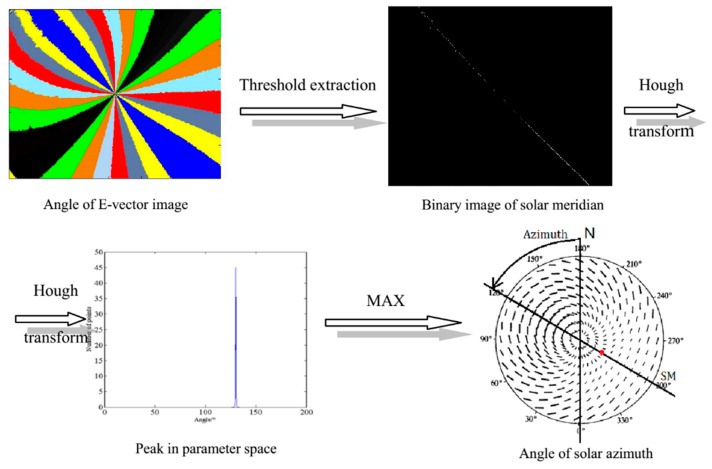
The flowchart of the orientation algorithm.

The peak in the parameter space is the angle of the solar meridian. The flow of the algorithm is shown in Figure 5.

## 5. Experimental Results

### 5.1. Calibration Results

**Figure 6:**
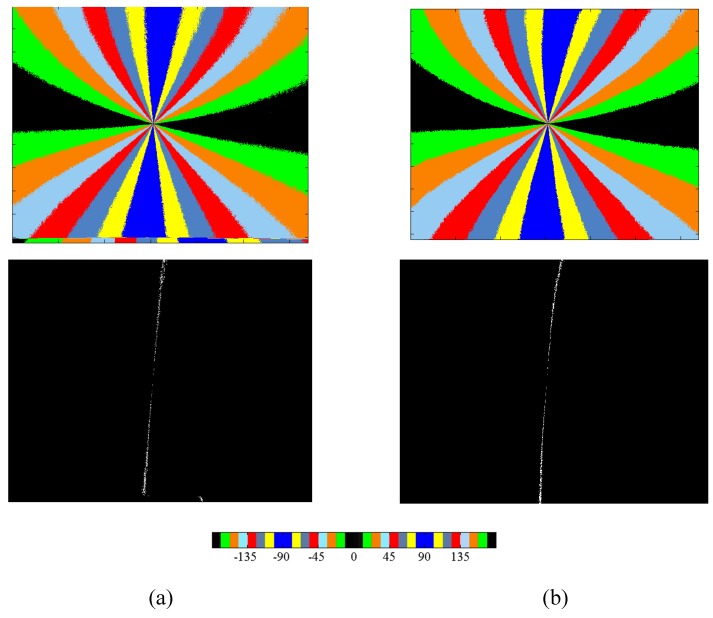
(**a**) ***AoE*** and ***BSM*** images calculated from the calibrated intensity images; and (**b**) ***AoE*** and ***BSM*** images calculated from the original intensity images.

We use the Matlab camera calibration toolbox, which contains Zhang’s calibration and stereo calibration, as well as a plane to calibrate the camera parameters. The three cameras simultaneously acquire images for extrinsic calibration, and the intrinsic parameters are calibrated. The intrinsic and extrinsic parameters are listed in Table 1. Figure 6 consists of the ***AoE*** image and the ***BSM*** image of solar meridian, which are original and calibrated.

**Table 1:** Measured intrinsic and extrinsic parameters of the cameras.

	Camera A	Camera B	Camera C
Intrinsic parameters			
Principal point	(616.68, 510.45)	(651.25, 560.96)	(643.76, 525.58)
Focal length	(1622.43, 1621.89)	(1619.19, 1619.91)	(1616.81, 1616.49)
Distortion coefficient	[−0.104 0.125]	[−0.091 0.059]	[−0.092 0.032]
Extrinsic parameters			
	[0 0 0]	[−0.014 0.013 −0.002]	[−0.014 0.0150.002]

Differences exist in the camera parameters, which cause serious errors and, thus, should be corrected. For example, the size of the CMOS sensor of the camera is 1024 × 1280 pixels, and their ideal principal points are (639.5, 511.5). According to Table 1, several dozens of pixels of difference exist between the measured and ideal principal points among the three cameras. According to Figure 6, the ***AoE*** and ***BSM*** images calculated from the original images have a certain degree of distortion, the binary image of solar meridian is insignificantly distorted, and the images calculated from the calibrated images are more similar to the ideal pattern in polarized skylight.

### 5.2. Orientation Experiment

**Figure 7:**
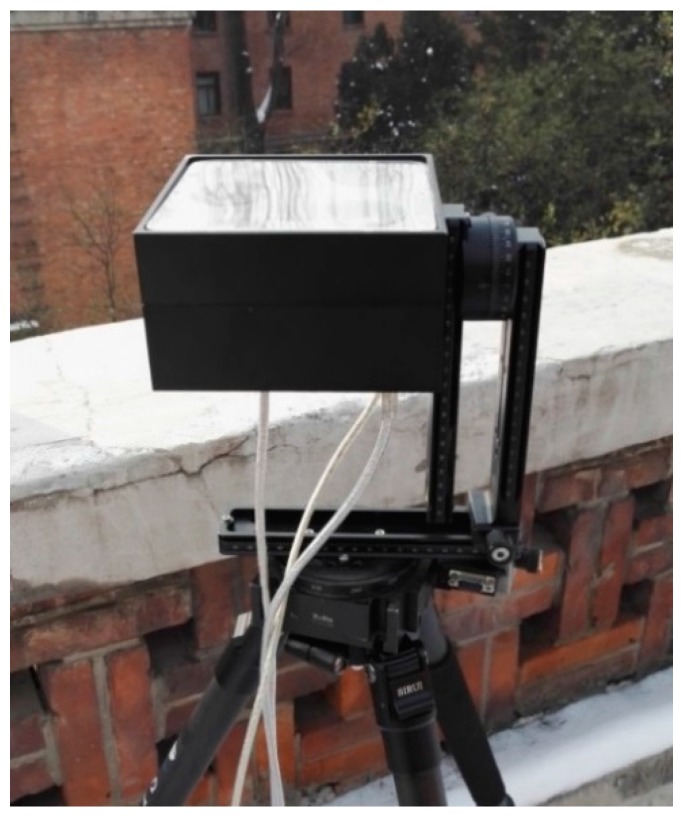
Image acquisition unit of the system.

**Figure 8:**
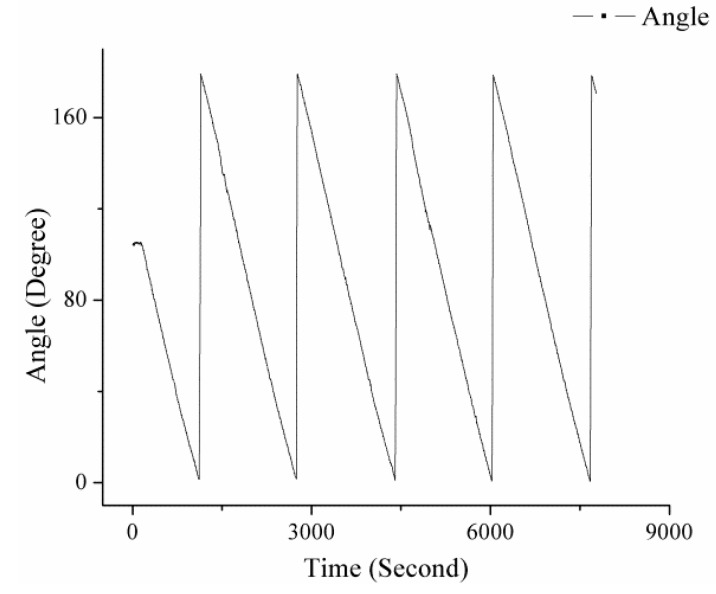
Angle measured throughout the system, which is uniformly rotated at a rate of 1° per second.

**Figure 9:**
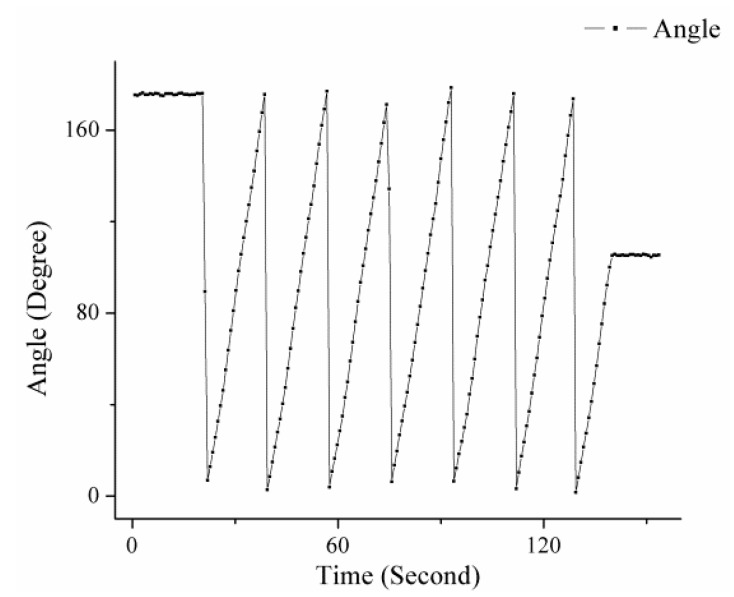
Angle measured throughout the system, which is uniformly rotated at a rate of 10° per second.

The experiment was conducted on 16 September 2015 at the top of a building located at 40° 0’ 22” N latitude and 116° 19’ 9” E longitude. The facilities besides the system are the motorized rotation stage (RSA100 Zolix), a controller (SC300-B Zolix), a battery (12 V 60 Ah), and a DC-to-AC inverter. The repeatability accuracy of the rotation stage system is better than 0.005°. The image acquisition unit of the system in experiment is shown in Figure 7. The rotation stage runs at speeds of 1° per second and 10° per second to verify the dynamic performance of the algorithm. The results are shown in Figure 8 and Figure 9.

According to the results, the algorithm that is used in the system can recongnize the dynamic polarized skylight pattern and solve the orientation from the pattern. The curves show that the system can track the motion of the rotation stage in rates of 1° per second and 10° per second.

Several groups of data are acquired to analyze the measurement error, as shown in Table 2 and Table 3. The error indicates the precision of the system and the algorithm. Twelve groups are acquired every 15° in the overall range (180°). The error is over 0.313°, and the population standard deviation is 0.148° ($\mathrm{population}\;\sigma^{2}=\sum_{group=1}^{12}N_{group}\sigma_{group}^{2}/\sum_{group=1}^{12}N_{group}$).

**Table 2:** Data and statistics of precision test Groups 1–6.

Group	1	2	3	4	5	6
Index	Data					
1	10.480	25.150	39.738	53.545	68.600	84.390
2	10.520	25.325	39.394	53.535	68.707	84.198
3	10.413	25.350	39.621	53.442	68.779	84.080
4	10.692	25.325	39.629	53.532	68.750	83.950
5	10.660	25.467	39.358	53.250	68.850	83.839
6	10.717	25.375	39.439	53.324	69.040	84.105
7	10.600	24.950	39.369	53.448	68.792	83.867
8	10.600	25.517	39.621	53.313	68.667	84.242
9	10.717	25.167	39.425	53.455	68.808	84.023
10	10.530	25.183	39.550	53.332	68.575	84.077
11	10.660	25.400	39.421	53.362	67.653	84.050
12	10.575	25.183	39.330	53.593	68.418	84.183
13	10.640	25.325	38.983	53.513	68.428	84.196
14	10.520	25.350	39.075	53.543	68.535	84.215
Statistics						
Mean	10.595	25.291	39.425	53.442	68.614	84.101
Σ	0.089	0.143	0.201	0.104	0.313	0.146

## 6. Conclusions and Outlooks

In this study, an orientation determination system was designed and implemented to verify and test the imaging polarization navigation algorithm. The system comprised three cameras to detect the polarized skylight. The essential calibration procedures, camera parameter calibration, and inconsistency of the response of CMOS were discussed, designed, and calibrated. The multi-camera system was easy to build because of the application of the three 8 mm lenses and the convenient calibration procedures for it. The calibration results were used to correct and rectify the images acquired by the cameras. The orientation determination experiment was conducted at different rates. The result indicated that the system could acquire and compute the polarized skylight images and resolve the orientation by the algorithm for verification in real-time. The update rate of the system was over 1 Hz, the error was over 0.313°, and the population standard deviation was 0.148° without any data filter.

The easy-to-build orientation determination experimental system enabled us to verify the polarization orientation algorithm in real time. However, certain problems remain. Synchronization is critical in control and data fusion in navigation. Thus, a synchronization signal should be involved for acquisition. Meanwhile, a heavy computational load is needed to acquire and process images for the PC in the system. Introducing a graphic processing unit and a digital signal processor will solve the problem and improve the measurement rate. Althouth significant polarization information and image information brings the advantages of high-precision and anti-interference, excessive redundant data would also increase computational load. Optimized data acquisition or an object tracking algorithm, such as a Kalman filter and mean shift, could focus on the key pattern in images instead of searching in entire image to decrease computational load. Finally, a bio-inspired polarization sensor for navigation must be added into an integrated navigation system that contains GNSS, INS, and magnetic sensors. Thus, the data fusion algorithm for polarization sensors is a significant development direction.

**Table 3:** Data and statistics of precision test Groups 7–12.

Group	7	8	9	10	11	12
Index	Data					
1	99.691	115.710	129.540	141.860	156.760	171.980
2	99.733	115.680	129.710	141.810	156.640	172.070
3	99.743	115.520	129.770	141.970	156.710	171.850
4	99.811	115.670	129.630	141.790	156.820	171.840
5	99.871	115.560	129.590	141.690	156.780	171.920
6	99.647	115.640	129.530	141.780	156.760	171.880
7	99.797	115.790	129.660	141.830	156.600	172.040
8	99.629	115.810	129.560	141.820	156.810	172.060
9	99.546	115.990	129.540	141.850	156.530	172.020
10	99.664	115.630	129.800	141.790	156.580	171.920
11	99.648	115.750	129.490	141.730	156.440	171.910
12	99.763	115.760	129.800	141.900	156.660	171.960
13	99.586	115.680	129.650	141.850	156.720	171.750
14	99.708	115.780	129.990	141.870	156.640	171.820
Statistics						
Mean	99.703	115.712	129.661	141.824	156.675	171.930
Σ	0.087	0.112	0.134	0.067	0.107	0.093
